# Pharmacology education and antibiotic self-medication among medical students: a cross-sectional study

**DOI:** 10.1186/s13104-017-2688-4

**Published:** 2017-07-27

**Authors:** Devarajan Rathish, Buddhika Wijerathne, Sandaruwan Bandara, Susanhitha Piumanthi, Chamali Senevirathna, Channa Jayasumana, Sisira Siribaddana

**Affiliations:** 1 Department of Pharmacology, Faculty of Medicine and Allied Sciences, Rajarata University of Sri Lanka, Saliyapura, Sri Lanka; 2 Department of Community Medicine, Faculty of Medicine and Allied Sciences, Rajarata University of Sri Lanka, Saliyapura, Sri Lanka; 3 Department of Medicine, Faculty of Medicine and Allied Sciences, Rajarata University of Sri Lanka, Saliyapura, Sri Lanka

**Keywords:** Pharmacology, Medical education, Self-medication, Medical undergraduates, Antibiotics

## Abstract

**Objective:**

Pharmacology teaches rational prescribing. Self-medication among medical students is recognised as a threat to rational prescribing. Antibiotic self-medication could cause antibiotic resistance among medical students. We aimed to find an association between pharmacology education and antibiotic self-medication.

**Results:**

Overall, 39% [(110/285) 95% CI 32.9–44.3] of students were found to have antibiotic self-medication. The percentage for antibiotic self-medication progressively increased with the year of study. The percentage of antibiotic self-medication was significantly high in the “Formal Pharmacology Education” group (47%—77/165) in comparison to the “No Formal Pharmacology Education” group (28%—33/120) (P = 0.001032). Overall, the most common self-prescribed antibiotic was amoxicillin (56%—62/110).

## Introduction

Medical education uses a wide range of teaching methodologies [1]. In Sri Lanka (SL) medical students read for a Bachelor of Medicine and Bachelor of Surgery (MBBS) degree similar to United Kingdom [2]; Pharmacology is learnt in 3rd and 4th years and includes rational prescribing and legal provisions involved in prescribing and dispensing [3, 4]. After obtaining the MBBS degree it is mandatory to complete a 1-year internship to obtain registration at the Medical Council. This registration provides the legal mandate to prescribe. No pharmacist can dispense prescription-only drugs without a valid prescription from a doctor. Despite these regulations, dispensing without a prescription, self-medication and prescribing by unqualified are common.

World Health Organization (WHO) defines self-medication as *“use of pharmaceutical or medicinal products by the consumer to treat self*-*recognized disorders or symptoms, the intermittent or continued use of a medication previously prescribed by a physician for chronic or recurring disease or symptom, or the use of medication recommended by lay sources or health workers not entitled to prescribe medicine”* [5]. Self-reliance, convenience and low cost are perceived benefits of self-medication. Potential risks are incorrect diagnosis, delay or failure to seek medical advice, risk of adverse effects, development of resistant microbes and incorrect prescribing [5]. A Spanish study revealed females, students, lonely people, urban dwellers, people with better education and aged more than 40 are more likely to self medicate [6].

A meta-analysis in 2014 showed 4–92% prevalence of self-medication among adolescents in South American and European countries [7]. Antibiotic self-medication has been recorded among university students; 44% in Romania [8], 13% in Sri Lanka [9] and 39% in Nigeria [10]. Another meta-analysis (2015) showed 38.8% (95% CI 29.5–48.1%) antimicrobial self-medication in low and middle-income countries [11]. Symptoms related to infections of respiratory, gastro-intestinal, skin, eye and ear, and urinary systems, and malaria are the reasons for self-medication; medicines commonly used are antibacterials and antimalarials. Representatives of pharmaceutical companies, pharmacists, relatives, friends and drug leaflets were the most common sources of information. Past successful use is a reason for repeated self-medication. Antimicrobial drugs were obtained from pharmacies, hospitals, leftovers from earlier purchase and friends [11].

A Sri Lankan study reported a significantly higher percentage of self-medication in an urban district [12.2%] compared to a rural district [7.9%] [12]. Another study revealed amoxicillin (95.4%) as the most common antibiotic and the upper respiratory tract infection (84.9%) as the most common illness related to self-medication [9].

According to WHO, antibiotic resistance is defined as “*resistance of a microorganism to an antimicrobial drug that was originally effective for treatment of infections caused by it”.* This leads to hampered infection control, increased cost and mortality. Although the evolution of resistant strains is a natural phenomenon, certain human actions accelerate its emergence and spread. WHO requests to use antimicrobial drugs, only when prescribed by a doctor [13].

Pharmacology education could encourage antibiotic self-medication among students. Medical students are exposed to infection during their clinical work and are at higher risk of infections. We assessed the association between pharmacology education and antibiotic self-medication practices among undergraduates.

## Main text

### Methods

A descriptive cross-sectional study was conducted from March to July 2016 among undergraduates of Faculty of Medicine and Allied Sciences, Rajarata University of Sri Lanka, Anuradhapura. Anuradhapura is 210 km away from the capital of Sri Lanka (Colombo). It has a population of nearly 856,500 [14]. 94.6% belong to the rural sector [14]. Agriculture is their main occupation (55%) [15]. The teaching hospital is situated 10 km away and the nearest pharmacy is located 5 km away from the faculty. The State Pharmaceutical Corporation’s retail sales outlet and several other private pharmacies are located closer to the Teaching Hospital.

All students of the faculty were approached and 696 out of 902 gave consent. First and second-years were categorised into No Formal Pharmacology Education (NFPE) group and third, fourth and fifth-years were categorised into students with Formal Pharmacology Education (FPE). The posthoc calculation of power was 90.9% (parameters used: percentage of antibiotic self-medication among FPE = 47%, percentage among NFPE = 28%, number of subjects in FPE = 165, number of subjects in NFPE = 120, margin of error = 5%).

We used a self-administered questionnaire to collect data on demography and antibiotic self-medication. The questions on antibiotic medication were adapted from a pre-validated questionnaire which was used in Jordan, with permission [16]. Question number 6 was modified from “What was the name of this antibiotic?” to “What were the names of these antibiotics?”. Parents occupation was classified according to International Standard Classification of Occupations [17].

The questionnaire, information sheet and consent form were given to students by trained MBBS qualified data collectors. Students were expected to leave back the questionnaires (filled/blank) and the consent forms on their seats after the lecture. To maintain confidentiality and prevent coercion, permanent lecturers at the faculty were not involved in describing, obtaining consent or collection of the questionnaires. First- and second authors analysed data using Microsoft Excel. Percentages were used to describe the demography and prevalence of antibiotic self-medication. Chi square test was used to find a significance of antibiotic self-medication between the two study groups (FPE vs NFPE).

### Results

Participation rate was satisfactory [77% (696/902)] with 86% (154/180) in the first year, 94% (172/183) in the second year, 61% (110/181) in the third year, 68% (125/185) in the fourth year and 78% (135/173) in the fifth year. NFPE category had 47% students (326/696). Mean age of the participants was 23.2 (SD ±1.6) years. 71% (491/696) were females. The majority of the participants were Buddhist (85%) and from the district of Gampaha (15%). 50% were staying near the faculty, 44% near the Teaching Hospital and the rest were travelling from home. Fathers of students were mostly “professionals” (37%) whereas the mothers were housewives (60%).

During last month, 40.9% [(285/696) 95% CI 37.3–44.6%] have taken antibiotics and 165 were students with FPE. Of them 39% [(110/285) 95% CI 32.9–44.3%] self-medicated with increasing number self-medicating as they progress through the medical faculty; 23% (14/60) students were from the first year, 33% (19/57) from second year, 39% (23/59) from third year, 46% (25/54) from fourth year and 53% (29/55) from fifth year. Students with FPE (70%; 77/110) were the majority among those who self-medicated with antibiotics. Self-medication was significant among students with FPE (47%—77/165) compared to students with NFPE (28%—33/120) (Chi square statistic = 10.7689; P = 0.001032).

Among those who had antibiotics, 70% (200/285) were females. They were the majority among all those who self-medicated (57%—63/110). However, self-medication was significantly high among males (55%—47/85) compared to females (32%—63/200) (Chi square statistic = 14.2496; P = 0.00016).

A sore throat (46%—51/110) was the most common symptom for antibiotic self-medication. Previous experience of using the same drug was the most common reason for antibiotic self-medication (89%—98/110). Drugs have been obtained from retail pharmacies 82% (90/110), relatives or friends (14%—15/110) and leftover drugs at home (6%—5//110). Amoxicillin was the main self-prescribed antibiotic (56%—62/110). The dose was based on previous knowledge (45%—49/110). The majority of the participants have taken the antibiotic for 1–3 days (68%—75/110).

Of students who self medicate with FPE, most lived near the Teaching Hospital (91%—70/77) and with NFPE most lived near the faculty (91%—30/33). Symptoms related to upper respiratory tract were the most common illness associated with antibiotic self-medication in both groups (NFPE—72% and FPE—85%) (Table 1).

**Table 1:** Symptoms for which antibiotic self-medication was used

Symptoms	% (n = 33)	Symptoms	% (n = 77)
*NFPE group*		*FPE group*	
Runny nose	30	Sore throat	60
Flu	21	Runny nose	12
Sore throat	15	Flu	10
Abscess	12	Diarrhoea	09
Toothache	10	Toothache	04
Tonsil infection	06	Sinusitis	03
Ear infection	06	Acne	01
		Oral ulcers	01
Total	100	Total	100

*NFPE* No Formal Pharmacology Education, *FPE* Formal Pharmacology Education

Previous experience is the most common explanation for antibiotic self-medication given by the students with and without FPE (97% vs 86%) (Table 2). Retail pharmacies were the most common source of supply for antibiotics in both NFPE and FPE (Table 2). 1–3 days was the usual duration of antibiotic usage in both NFPE and FPE groups (Table 2). Amoxicillin was the main self-prescribed antibiotic in both NFPE and FPE groups, followed by ciprofloxacin in NFPE and co-amoxiclav in FPE (Table 3).

**Table 2:** Features of antibiotic self-mediation in NFPE and FPE group

NFPE group	% (n = 33)	FPE group	% (n = 77)
*Reasons for self-medication*			
Previous experience	97	Previous experience	86
No access to physician care	03	No access to physician care	06
		Poor economic status	04
		No specific reason	04
*Source of antibiotic supply*			
Retail pharmacy	79	Retail pharmacy	83
Relatives and friends	18	Relatives and friends	12
Household	03	Household	05
*Source of information on the use of the antibiotic*			
Previous knowledge	28	Previous knowledge	52
Physician	24	Physician	22
Pharmacist	24	Leaflet	09
Relative or friend	24	Relative or friend	05
		British National Formulary	05
		Pharmacist	04
		Internet	03
*Duration of antibiotic usage in each episode of self-medication (days)*			
1–3	85	1–3	61
4–7	12	4–7	34
>7	03	>7	05

*NFPE* No Formal Pharmacology Education, *FPE* Formal Pharmacology Education

**Table 3:** Top 5 antibiotics used in self-medication

Antibiotics^a^	% (n = 33)	Antibiotics^a^	% (n = 77)
*NFPE group*		*FPE group*	
Amoxicillin	58	Amoxicillin	56
Ciprofloxacin	06	Co-amoxiclav	21
Tetracycline	06	Azithromycin	12
Co-amoxiclav	06	Ciprofloxacin	12
Cefalexin	03	Cefalexin	08

*NFPE* No Formal Pharmacology Education, *FPE* Formal Pharmacology Education

^a^A participant could name more than one antibiotic, therefore total of percentage can be >100

### Discussion

The percentage of antibiotic self-medication progressively increased with the number of years spent in the undergraduate education. However, few encouraging signs were present. Students with FPE used antibiotics for a longer period. This may indicate their knowledge on the half-life of drugs, steady state concentration and time dependent killing.

A runny nose and flu were among the top three symptoms in both groups (Table 1). This clearly indicates inappropriate antibiotic use for viral infections.

Studies from West Bengal [18], Bahrain [19], Belgrade [20], Southern China [21] and Netherland [22] have shown a positive association between pharmacology education and self-medication. This is compatible with the present study. A Spanish study found high self medication by females [6] contrary to our findings.

Previous studies have also shown that the most common symptom was sore throat [16, 19, 21, 23, 24], reason was previous positive experience [16, 25–28], the source of the drug was retail pharmacy [24, 29, 30], antibiotic was amoxicillin [8, 9, 16, 23, 24] and source of information was previous knowledge [16] with regards to antibiotic self-medication.

It is alarming to note that the retail pharmacies were the main source of antibiotic self-medication. The current regulations prohibit dispensing of prescription-only antibiotic without a valid prescription. However, there is a lack of monitoring and oversight of retail pharmacies.

Amoxicillin is a useful first-line antibiotic for many common infections. Rampant, irrational use leads to resistance. Ciprofloxacin, a quinolone, was among the top five self-medicated antibiotics for both the groups. Drugs from the quinolone group of antibiotics are reserved as a second-line drug for tuberculosis. Anuradhapura has a high incidence (30.8 per 10^5^) and death (3.5 per 10^5^) rates for tuberculosis [31]. Blind use of ciprofloxacin makes medical students vulnerable to drug-resistant tuberculosis.

Students with FPE stay near the Teaching Hospital with easy access to state and private pharmacies. This could be a potential confounding factor causing high prevalence of self-medication.

The findings of the study warrant the need to improve access to medical care, reform pharmacology teaching and monitor drug dispensing at retail pharmacies. These could reduce the amount of self-medication in this population.

## Limitations

Findings of this study cannot establish a causal association neither it could be generalised because this is a cross-sectional study conducted at one medical faculty. A nationwide study including all other medical faculties of SL would be appropriate. However, the findings are unique because this faculty is located in a rural area with fewer pharmacies around. Confounders of the study could be access to retail pharmacies, the occupation of the parents (parents being health care workers might predispose to self-medication) and inadequate knowledge of pharmacology and microbiology among the students of FPE group. Exclusion of the above confounders is methodologically challenging.

## Abbreviations

FPE: Formal Pharmacology Education
MBBS: Bachelor of Medicine, Bachelor of Surgery
NFPE: No Formal Pharmacology Education
SL: Sri Lanka
WHO: World Health Organization
